# SIRT1 affects DNA methylation of polycomb group protein target genes, a hotspot of the epigenetic shift observed in ageing

**DOI:** 10.1186/s40246-015-0036-0

**Published:** 2015-06-24

**Authors:** Luisa A Wakeling, Laura J Ions, Suzanne M Escolme, Simon J Cockell, Tianhong Su, Madhurima Dey, Emily V Hampton, Gail Jenkins, Linda J Wainwright, Jill A McKay, Dianne Ford

**Affiliations:** Institute for Cell and Molecular Biosciences, Human Nutrition Research Centre, Newcastle University Medical School, Newcastle upon Tyne, NE2 4HH UK; Faculty of Medical Sciences, Newcastle University Medical School, Newcastle upon Tyne, NE2 4HH UK; Institute of Health and Society, Human Nutrition Research Centre, Newcastle University Medical School, Newcastle upon Tyne, NE2 4HH UK; Unilever R&D, Colworth Discover, Colworth Science Park, Sharnbrook, Bedfordshire, MK44 1LQ UK

**Keywords:** Dietary restriction, Polycomb group proteins, Polycomb repressive complexes, Stem cells

## Abstract

**Background:**

SIRT1 is likely to play a role in the extension in healthspan induced by dietary restriction. Actions of SIRT1 are pleiotropic, and effects on healthspan may include effects on DNA methylation. Polycomb group protein target genes (PCGTs) are suppressed by epigenetic mechanisms in stem cells, partly through the actions of the polycomb repressive complexes (PRCs), and have been shown previously to correspond with loci particularly susceptible to age-related changes in DNA methylation. We hypothesised that SIRT1 would affect DNA methylation particularly at PCGTs. To map the sites in the genome where SIRT1 affects DNA methylation, we altered SIRT1 expression in human intestinal (Caco-2) and vascular endothelial (HuVEC) cells by transient transfection with an expression construct or with siRNA. DNA was enriched for the methylated fraction then sequenced (HuVEC) or hybridised to a human promoter microarray (Caco-2).

**Results:**

The profile of genes where SIRT1 manipulation affected DNA methylation was enriched for PCGTs in both cell lines, thus supporting our hypothesis. SIRT1 knockdown affected the mRNA for none of seven PRC components nor for DNMT1 or DNMT3b. We thus find no evidence that SIRT1 affects DNA methylation at PCGTs by affecting the expression of these gene transcripts. EZH2, a component of PRC2 that can affect DNA methylation through association with DNA methyltransferases (DNMTs), did not co-immunoprecipitate with SIRT1, and SIRT1 knockdown did not affect the expression of EZH2 protein. Thus, it is unlikely that the effects of SIRT1 on DNA methylation at PCGTs are mediated through direct intermolecular association with EZH2 or through effects in its expression.

**Conclusions:**

SIRT1 affects DNA methylation across the genome, but particularly at PCGTs. Although the mechanism through which SIRT1 has these effects is yet to be uncovered, this action is likely to contribute to extended healthspan, for example under conditions of dietary restriction.

**Electronic supplementary material:**

The online version of this article (doi:10.1186/s40246-015-0036-0) contains supplementary material, which is available to authorized users.

## Background

The DNA methylation profile of the vertebrate genome changes over time, reflected as a change in total methyl-cytosine content [1, 2]. These changes have been mapped to specific sites in species including mice [3, 4] and humans [5–8], revealing a drift in DNA methylation across most of the genome with components that are both tissue specific and tissue independent [4, 9]. A notable feature of the age-related drift in DNA methylation is that hypermethylation clusters at the gene targets of polycomb group proteins (PCGTs), as observed in human whole blood from postmenopausal women [7], mouse intestine [4] and mouse haematopoietic stem cells [3]. Several arguments and observations support the premise that epigenetic changes, such as changes in DNA methylation, contribute to the ageing process. For example, the fundamental role of epigenetic reprogramming in the process of gamete formation, which must reverse the ageing clock to prevent progressively shortened lifespan in each successive generation, provides a compelling argument to support this view; likewise the role of epigenetic reprogramming to restore pluripotency in the success of somatic cell nuclear transfer [10]. Also consistent with the premise that epigenetic changes contribute to ageing is that extended lifespan can be inherited trans-generationally in *Caenorhabditis elegans* via genes that are components of a major epigenetic modifier—the histone H3 lysine 4 trimethylation (H3K4me3) complex [11]. The polycomb group proteins bind to PCGTs as polycomb repressive complexes (PRCs). PCGTs are repressed by mechanisms involving chromatin modification in stem cells and must be expressed to achieve cell differentiation [12]. PCGTs also tend to be hypermethylated in cancer [13–15].

We showed recently that manipulating the expression of the histone deacetylase SIRT1 in human cells affected promoter DNA methylation of a small panel of genes that we tested, selected on the basis that they have been reported to show an age-related change in DNA methylation and to be expressed differentially in response to dietary restriction (DR), an intervention shown robustly in multiple species to increase lifespan and/or healthspan [16]. The view that SIRT1 contributes to increased healthspan and/or lifespan, including under conditions of DR, is controversial. The supporting literature is extensive and is covered by recent reviews (e.g. [17, 18]). Notable recent developments include the observation that male and female transgenic mice that overexpress Sirt1 specifically in the brain had extended lifespan and enhanced neural activity in the dorsomedial and lateral hypothalamic nuclei [19]. It appears, however, that some earlier work in model organisms proposed to demonstrate that the gene homologues of SIRT1 confer extended lifespan requires re-evaluation. For example, extended lifespan in strains of *C. elegans* transgenic for *Sir2* tracked with loci other than the transgene [20]. Also, confounding effects of genetic manipulation used to create *Sir2* transgenic *Drosophila*, rather than the *Sir2* transgene per se, appear to be responsible for the long-lived phenotype [20]. However, the debate has been re-opened by reports including that lifespan was extended in *Drosophila* when *Sir2* expression was manipulated using an inducible system that eliminated genetic background as a confounding factor [21]. Also, a body of other recent data show consistently effects on mammalian physiology commensurate with sirtuins having actions that protect against features of ageing (reviewed in [22]). Intermediates in pleiotropic cellular pathways and several key transcription factors with likely effects on healthspan are substrates for deacetylation by SIRT1. These substrates include PGC1α, which controls mitochondrial biogenesis, p53 [23] and many others [24]. Our discovery that SIRT1 affects DNA methylation with a bias towards genes that also show altered expression in response to dietary restriction [16] uncovers a novel and fundamental function of SIRT1 with likely particular relevance to its effects on healthspan. Recent reviews provide a fuller exposition of evidence supporting the view that SIRT1 has a role in healthspan (e.g. [25]).

Here we hypothesised that altering the level of SIRT1 expression would affect DNA methylation on a genome-wide basis and target preferentially genes, including PCGTs, where DNA methylation is affected by increasing age. Supporting our hypothesis, we made the fundamentally important observation that effects of SIRT1 on DNA methylation do indeed cluster particularly at PCGTs.

## Results

### Manipulating SIRT1 expression affects DNA methylation across the genome

**Fig. 1:**
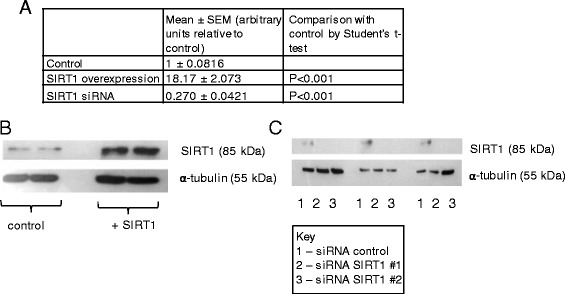
Confirmation of SIRT1 overexpression and knockdown in HuVECs. **a** Measurement of SIRT1 mRNA by RT-qPCR. Data are for *n* = 4–8. Measurement of SIRT1 by Western blotting following transient transfection with plasmid pCMV6-ENTRY-SIRT1 (Origene) or with vector control (**b**) or with siRNA targeted to SIRT1 or with control siRNA (**c**). Approximately 10 μg of protein was loaded in each lane. Three biological replicates are presented for each condition. Approximate molecular weights are indicated. “+SIRT1” indicates cells transfected with pCMV6-ENTRY-SIRT1; “control” indicates cells tranfected with vector control; “siRNA” indicates cells transfected with one of two siRNAs (#1 or #2) targeted to SIRT1 or with a control siRNA. Data are representative of multiple independent repeats of the procedure

We increased SIRT1 expression by transient transfection with a plasmid construct or reduced expression using siRNA (as in our previous work [16]) to measure the effect on DNA methylation across the genome in two different human cell line models—HuVECs (vascular endothelial) and (as used in our previous work) Caco-2 (intestinal) cells. Efficacy of overexpression or knockdown for HuVECs was confirmed by RT-qPCR and Western blotting (Fig. 1) and has been shown previously for Caco-2 cells [16]. DNA was enriched for the methylated component and compared to the input sample. For HuVECs, a recombinant H6-GST-MBD protein was bound to fragmented DNA, and then the methylated fraction was captured on magnetic beads coated with GSH. Input and enriched samples were then sequenced, and reads were mapped to the human genome then filtered to those within 2 kb of a transcription start site or within genes (between the TSS and stop codon). The data are deposited under GEO accession number GSE54072 [26]. Differentially methylated genes were identified using the package MEDIPS (Bioconductor) then classified as hypomethylated or hypermethylated when SIRT1 expression was increased or reduced. For Caco-2 cells, DNA was enriched for the methylated fraction by MeDIP using an antibody recognising 5-methylcytidine (5mC), and efficacy was confirmed by measuring enrichment by qPCR of a lambda phage DNA added as a spike to all samples in both a demethylated and in vitro-methylated form and of two loci known to be hypermethylated (*H19* and *L1.2*) relative to a hypomethylated locus (*UBE2B*) [27]. Comparing input and immunoprecipitated samples, the lambda phage spike was enriched 1000–12,000-fold and the endogenous hypermethylated versus hypomethylated loci were enriched 40–270-fold (see Additional file 1), thus confirming efficacy. Input and enriched samples were co-hybridised to the human 3x720K CpG Island Plus RefSeq Promoter Array (NimbleGen). The data are deposited under GEO accession number GSE53569 [28]. Genes were scored as methylated differently under conditions of SIRT1 knockdown compared with control where they appeared only in one or other list of enriched genes.

**Fig. 2:**
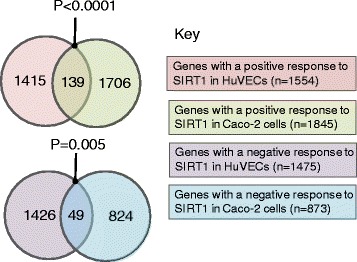
Intersections between lists of genes that showed positive or negative DNA methylation responses to SIRT1. Data are shown for HuVECs and Caco-2 cells, as defined in the key. *P* values were derived using chi-square analysis applying Yates’ correction

For ease of reference, we refer to genes that lost DNA methylation with SIRT1 knockdown and/or gained DNA methylation with SIRT1 overexpression as having a positive DNA methylation response to SIRT1. Conversely, we classify genes that responded to SIRT1 in an opposite direction as having a negative response to SIRT1. A total of 1554 genes in HuVECs [29] and 1845 genes in Caco-2 cells [29] showed a positive DNA methylation response to SIRT1[29], of which 139 (a larger number than expected by chance; *P* < 0.001) were common to both cell lines (Fig. 2). Similarly, the two different cell lines shared a subset of genes that showed a negative DNA methylation response to SIRT1 that was greater than expected by chance (*P* = 0.005), comprising 49 genes from a total of 1475 in HuVECs [29] and 873 in Caco-2 cells [29] (Fig. 2).

### PCGTs are over-represented among genes for which DNA methylation is affected by SIRT1 manipulation

We determined if PCGTs were over-represented in our list of genes that responded to SIRT1 using lists derived by performing genome-wide location analysis of DNA immunoprecipitated by antibodies against core components of polycomb repressive complex 1 (PRC1) (Phc1 and Rnf2) and PRC2 (Suz12 and Eed) [30]. This analysis is summarised in Table 1. Gene targets of each individual component of PRC1 or PRC2, or targets of at least one component, were all enriched 1.3 to 1.8-fold in genes we found to show a positive DNA methylation response to SIRT1 in both cell lines (with the exception of targets of Rnf2 in HuVECs). Similarly, genes identified as targets of ALL components of PRC1 and PRC2 were enriched (1.6 to 2.2-fold) in the genes showing a positive DNA methylation response to SIRT1. We found a similar relationship between genes that showed a negative DNA methylation response to SIRT1 and PCGTs. Gene targets of each individual component of PRC1 or PRC2 as well as gene targets of at least one component were all enriched 1.3 to 1.7-fold in these gene lists derived from both cell lines (with the exception of Eed in HuVECs). Genes identified as targets of ALL components of PRC1 and PRC2 [30], however, were not enriched in the lists of genes that responded negatively to SIRT1.

**Table 1:** Analysis of the size of intersections between polycomb group protein target genes (PCGTs) and genes with higher levels of DNA methylation

	HuVEC				Caco-2			
	Positive response to SIRT1		Negative response to SIRT1		Positive response to SIRT1		Negative response to SIRT1	
	RF	*P*	RF	*P*	RF	*P*	RF	*P*
Suz12 targets	1.5	<0.0001	1.5	=0.0008	1.6	<0.0001	1.5	=0.0088
Eed targets	1.4	=0.0102	*1.1*	*=0.4573*	1.8	<0.0001	1.5	=0.0347
Phc1 targets	1.4	=0.0069	1.3	<0.0001	1.6	<0.0001	1.7	=0.0004
Rnf2 targets	*1.2*	*=0.0853*	1.3	=0.0341	1.7	<0.0001	1.7	=0.0012
Targets of all polycomb group proteins	1.6	<0.0001	*1.1*	*=0.6135*	2.2	<0.0001	*1.4*	*=0.1355*
Targets of at least one polycomb group protein	1.3	=0.0090	1.3	=0.0046	1.5	<0.0001	1.6	<0.0001

Analysis of the size of intersections between polycomb group protein target genes (PCGTs) and genes with higher levels of DNA methylation under control conditions and/or reduced levels of DNA methylation under conditions of SIRT1 knockdown (positive response to SIRT1) or genes with reduced levels of DNA methylation under control conditions and/or higher levels of DNA methylation under conditions of SIRT1 knockdown (negative response to SIRT1). PCGT lists were compiled from published data (Boyer et al. 2006). RF (representation factor) values show the ratio of observed to expected number of genes in the intersection. *P* values were derived using chi-square analysis applying Yates’ correction. Italicized cells indicate where data did not reach statistical significance

### Polycomb group protein mRNA levels are not affected by SIRT1 manipulation

**Fig. 3:**
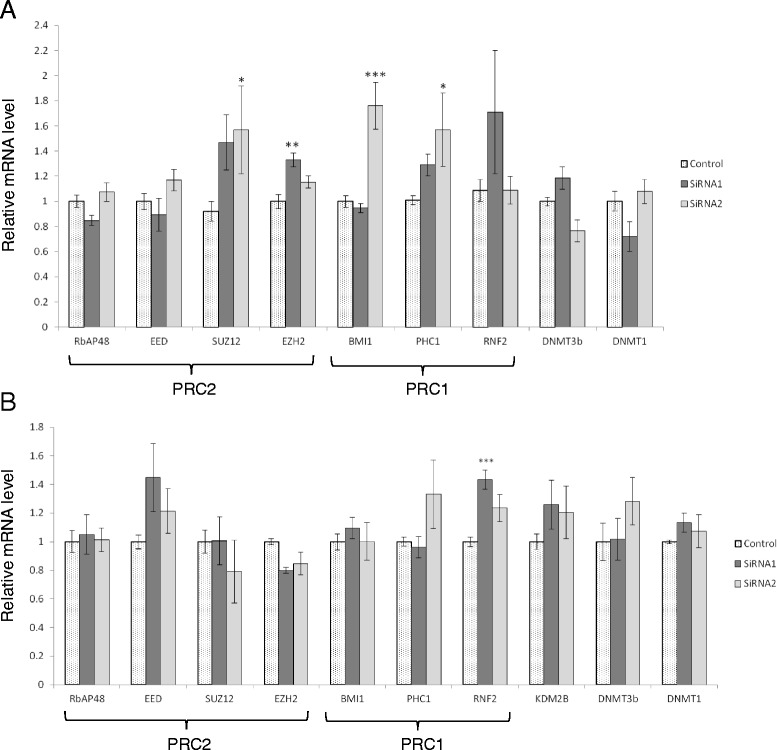
Measurement of mRNAs for PRC components, KDM2B and DNMTs under conditions of SIRT1 knockdown. Data are for HuVECs (**a**) and Caco-2 cells (**b**). SIRT1 knockdown was achieved using two different siRNAs, as indicated. Measurements were by RT-qPCR. Data are mean ± SEM for *n* = 6–15 relative to the reference genes GAPDH and TOP1 and normalised according to the measurements for the control siRNA. *P < 0.05; **P < 0.01; ***P < 0.001 compared with control by one-way ANOVA then Dunnett’s post hoc test

The chromatin modifications that repress PCGTs in stem cells result partly from actions of the polycomb group proteins themselves to effect epigenetic modification [31], including DNA methylation [32]. Thus, we proposed that SIRT1 may affect DNA methylation at PCGTs by changing the level of expression of polycomb group proteins. We investigated this hypothesis by determining the effect of SIRT1 knockdown in HuVECs and Caco-2 cells on the relative level of mRNA for individual polycomb group proteins (components of PRC1 and PRC2). We also determined if SIRT1 knockdown affected the mRNA for the histone demethylase KDM2B, which has been shown to recruit PRC1 to CpG islands [33]. None of the mRNAs measured was changed consistently when SIRT1 expression was reduced using both siRNAs (separately) (Fig. 3). Increases in SUZ12, EZH2, BMI1 and PHC1 mRNAs in HuVECs and in RNF2 mRNA in Caco-2 cells were observed using only one of the two siRNAs in each instance. Given that the second siRNA was equally effective in reducing SIRT1 expression, then these responses cannot be attributed to SIRT1 knockdown. Off-target effects of the siRNA on genes that influence the expression of these polycomb group protein mRNAs is a possible explanation for these observations. We thus found no evidence to support the idea that SIRT1 affects DNA methylation at PCGTs by effects on the expression of PRC components.

### SIRT1 does not affect the quantity of EZH2 protein in the cell nor associate with EZH2

**Fig. 4:**
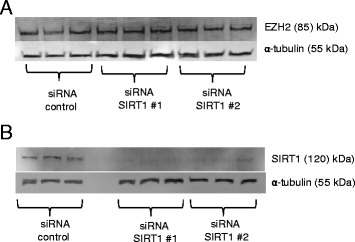
Measurement of EZH2 protein under conditions of SIRT1 knockdown. SIRT1 knockdown in Caco-2 cells was achieved using two different siRNAs and protein was analysed by Western blotting using anti-EZH2 antibody (**a**) or anti-SIRT1 antibody (to confirm efficacy of knockdown) (**b**). Blots were probed with anti-α-tubulin antibody to confirm equal protein loading and transfer. Approximately 10 μg of protein was loaded in each lane. Three biological replicates are presented for each condition. Approximate molecular weights are indicated

**Fig. 5:**
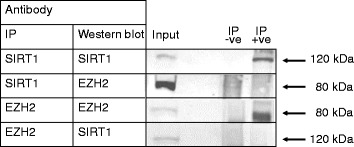
Use of anti-SIRT1 and anti-EZH2 antibodies in co-immunoprecipitation experiments. Anti-SIRT1 or anti-EZH2 antibody was added to total cell lysate as indicated, but omitted from samples labelled “IP -ve”. Immune complexes were captured on protein A/G Sepharose then eluted and analysed, along with an equivalent sample of the input protein, by Western blotting using anti-SIRT1 or anti-EZH2 antibody, as indicated. Both antibodies when used for immunoprecipitation and Western blotting confirmed self-enrichment of the corresponding protein in the immunoprecipitated samples but neither led to enrichment of the other protein. Approximate molecular weights of proteins seen as specific bands are indicated. Data using anti-SIRT1 antibody are for Caco-2 cells and are consistent with data obtained using HuVECs. Data using anti-EZH2 antibody are for HuVECs and are consistent with data obtained using Caco-2 cells

It has been shown that the histone methyltransferase EZH2 (a component of PRC2) associates with DNA methyltransferases (DNMTs) and is necessary to recruit DNMTs to EZH2-repressed genes [32]. Also, an intermolecular association between recombinant SIRT1 and EZH2 was observed in HeLa cells. [34]. We thus reasoned that effects on EZH2 were a likely point of action through which SIRT1 affects DNA methylation at PCGTs. To explore further if SIRT1 affects the expression of EZH2, we determined by Western blotting if SIRT1 knockdown affected EZH2 protein expression in Caco-2 cells and saw no effect (Fig. 4). We also investigated if EZH2 co-immunoprecipitated with SIRT1. We achieved successful immunoprecipitation of both SIRT1 and EZH2 from Caco-2 cells and HuVECs but detected no EZH2 in the protein fraction enriched using anti-SIRT1 antibody and no SIRT1 in the protein fraction enriched using anti-EZH2 antibody (Fig. 5). We thus found no evidence that SIRT1 and EZH2 form an intermolecular complex in these cell lines.

## Discussion

Our findings make an important contribution to key developments in understanding how age-related changes in DNA methylation contribute to the process of ageing, a field where the importance of PCGTs is just beginning to emerge. Salient points are that (1) the DNA methylation signatures of a mixed cell population from human blood during ageing and mouse intestinal cells mimicked features common to both stem cells and cancer with respect to PCGTs [4, 7]; (2) changes in DNA methylation during the ageing of haematopoietic stem cells clustered particularly at PCGTs [3]; (3) reversal of age-driven accumulation of DNA methylation changes in stem cells (“epigenetic rejuvenation”) may reverse the ageing process [10]. An effect of SIRT1 on DNA methylation at PCGTs is a highly novel and important finding and is pertinent to evaluating how the impact of SIRT1 on pleiotropic cellular processes may affect healthspan.

We made this fundamentally important discovery concerning actions of SIRT1 in two different cell lines using approaches based on different principles both to enrich DNA for the methylated fraction and for downstream detection of the enriched sequences. The discovery is thus highly robust, and we make no attempt to confirm effects by direct measurement of DNA methylation at specific PCGTs. In contrast to techniques we could apply for such measurements, neither approach we used reports on DNA methylation at specific CpG sites. Both approaches sample the total level of DNA methylation across a fragment of DNA, whereas approaches to targeted measurements (such as pyrosequencing, which we use routinely [16]), sample only a very limited number of CpG sites. Thus, failure of targeted approaches to validate findings based on genome-wide analysis may reflect sampling of unaffected CpG sites in the vicinity of affected sites. Despite these caveats, we did observe that 4 of a panel of 10 genes (*IRX3*, *PTPRG*, *STK10* and *KLF3*) we found previously to show differential DNA methylation when the expression of SIRT1 was manipulated in Caco-2 cells [16] were also detected as differentially methylated in the current analysis.

We reported previously the effect of SIRT1 knockdown in Caco-2 cells on the transcriptome [16]. Comparison of the list of genes that underwent DNA hypermethylation or hypomethylation in response to SIRT1 knockdown with the list of genes for which we detected a parallel change in expression revealed no significant correlation. This finding is consistent with a wider body of published data that reveals at best a weak correlation between effects on DNA methylation and gene expression. For example, correlation between age-related changes in genome-wide DNA methylation in haematopoietic stem cells, which clustered at genes regulated by PRC2, and changes in gene expression was low [3], suggesting that effects are manifest at the level of the transcriptome only when passed on to downstream progeny or indirectly. Furthermore, studies in diverse cell types have revealed that there is generally little correlation between changes in genome-wide DNA methylation and gene expression [35–37]. Reported weak correlations between DNA methylation and gene expression were more pronounced for cell lineage-specific genes where DNA methylation changes were in regulatory elements [3, 35]. The resolution of our current data does not allow the identification of DNA methylation changes that are specifically within regulatory elements. Thus, attempting to validate our data on SIRT1-driven effects on DNA methylation at PCGTs by measurement of the response at the RNA level of these genes would thus be of limited value.

A future priority should be to uncover in detail the mechanism through which SIRT1 affects DNA methylation at PCGTs. Action mediated through the polycomb group proteins is a highly plausible suggestion, given that the PRCs affect the epigenetic status, including DNA methylation, of PCGTs [31, 32]. We found no evidence that the level of SIRT1 in the cell affects expression at the mRNA level of any of the components of PRC1 or PRC2 or of KDM2B, which targets PRC1 to CpG islands [33]. Our data do not exclude the possibility that SIRT1 affects polycomb group protein expression downstream of mRNA, and thus, measurement of the effect of SIRT1 knockdown on polycomb group protein level (e.g. by Western blotting) should be a future priority. Of the multiple components of the PRCs, EZH2 is arguably the most likely candidate as the point at which SIRT1 interacts to modify actions of the PRCs on DNA methylation at their gene targets because EZH2 has been shown to control DNA methylation through association with DNMTs [32]. Also, direct intermolecular association between recombinant, epitope-tagged SIRT1 and EZH2 was observed in HeLa cells [34]. Moreover, trimethylation of H3K27 by EZH2 is an early event in the sequence of epigenetic modifications that results from PRC binding to PCGTs and leads to recruitment of PRC1 through chromodomain-containing components [31]. However, we detected no intermolecular association between SIRT1 and EZH2. The interaction observed in HeLa cells may require the expression of the two proteins at higher levels (as was the case in this previous work, by virtue of expression of recombinant proteins from transgenes) or may be cell-line specific. Moreover, the earlier work showed that SIRT1 is not a component of PRC2 but associates with polycomb proteins in PRC4, a specific PRC containing isoform 2 of EED [34]. EED2 is expressed specifically in undifferentiated pluripotent cells and also cancer cells. The same work showed a direct association between SIRT1 and SUZ12 using purified recombinant proteins. A priority for future work is to determine if SIRT1 interacts directly with other components of the PRCs and to determine by ChIP if SIRT1 binds directly to PCGTs or if its effects on DNA methylation at these sites are indirect.

**Fig. 6:**
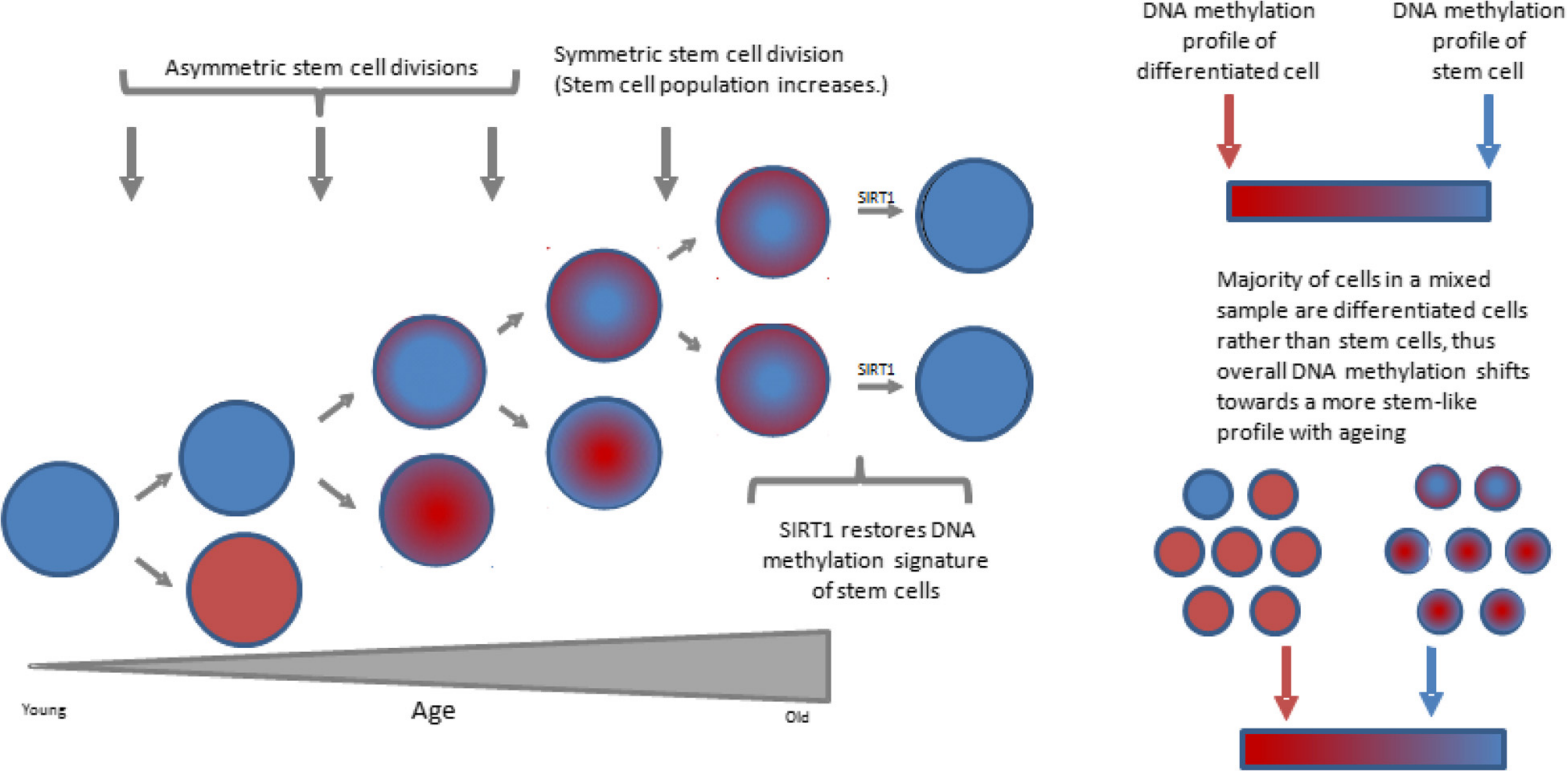
A proposed model for age-associated drift in DNA methylation and restorative effects of SIRT1. The stem cell, which has a characteristic DNA methylation signature (*blue*), divides asymmetrically to generate a differentiated daughter cell with a different DNA methylation signature (*red*) and a daughter stem cell. The fidelity with which the two daughter cells that arise from this asymmetric division acquire the correct pattern of DNA methylation across the genome is compromised in ageing tissue such that the DNA methylation pattern of the retained stem cell becomes more skewed towards that of the differentiated cell and vice versa, as indicated by the increasing “blending” of the colours representing the different DNA methylation signatures. Additionally, the proportion of stem cells may increase in ageing tissue (by symmetric stem cell division). The age-related drift in DNA methylation in a sample of stem cells, therefore, will be towards the signature of the differentiated cell. In a mixed cell population, however, where differentiated cells predominate, the drift in DNA methylation is towards the signature of the stem cell. Effects of SIRT1 on DNA methylation are to mimic the signature of the stem cell, rather than the differentiated cell; thus, beneficial effects on healthspan are via effects on stem cells, where the correct (“younger”) DNA methylation signature is restored, facilitating “corrected” partitioning of the DNA methylation signature at cell division, and thus restored tissue function

A factor to consider in interpreting the likely implications of age-associated changes in PCGT DNA methylation and the effects thereon of SIRT1 is the nature of the cell population sampled and/or analysed, specifically whether these be stem cells [3] or, principally, the differentiated progeny [4, 7]. Lack of stemness in stem cells or gain of stem cell-like features in the differentiated progeny could give rise to features of tissue ageing. We propose a speculative model, based on this premise, that can reconcile these observations on DNA methylation changes in ageing cells, including effects at PCGTs, with the observed effects thereon of SIRT1 being a counteracting mechanism (Fig. 6). We propose that the fidelity with which two daughter cells that arise from asymmetric stem cell division acquire the correct pattern of DNA methylation across the genome is compromised in ageing tissue. Viz, the DNA methylation pattern of the retained stem cell becomes more skewed towards that of the differentiated cell and vice versa. Epigenetic drift in a sample of ageing stem cells, therefore, will be towards the epigenome of the differentiated cell. In a mixed cell population, however, where differentiated cells predominate (including intestine [4], as used to compile our list, and leukocytes [7]), epigenetic drift will be towards the epigenome of the stem cell. The more stem-like DNA methylation signature reflects faltering of the differentiation process such that cells retain some of the signature of their stem progenitors. Additionally, or alternatively, as tissues age, the proportion of differentiated versus stem cells may shift towards there being a larger proportion of stem cells. Indeed, an expanded proliferative zone has been observed in the intestinal mucosa of older, compared with younger, rats and humans [38, 39], and the number of clonogenic cells per intestinal crypt on older mice, detected following irradiation damage, exceeded eightfold the number in younger mice [40]. We make the speculative suggestion that effects of SIRT1 on DNA methylation contribute to improved healthspan by restoring the DNA methylation profile of stem cells, thus enhancing tissue capacity to regenerate and restore function. We propose that SIRT1 has these actions through association with PRCs, which in turn modulates effects of these complexes on DNA methylation. Thus, establishing rigorously which polycomb proteins or combinations thereof co-immunprecipitate with SIRT1 and mapping the sites on the genome where these complexes bind are immediate future priorities.

## Conclusions

We show that effects of SIRT1 on DNA methylation cluster at PCGTs. There is already robust evidence that these loci are also hot spots for age-related changes in DNA methylation. The discovery thus advances substantially our understanding of how the pleiotropic effects of SIRT1 may contribute to healthspan. Future research should explore the mechanisms that account for these effects of SIRT1 on DNA methylation at PCGTs and how these actions then affect stem cell biology. Such knowledge will point towards actions of SIRT1 whose mimicry by lifestyle or pharmaceutical interventions may contribute to a longer, healthier life.

## Methods

### Cell culture

Caco-2 cells were cultured under our standard laboratory conditions as described previously [41]. HuVECs (passage 3) were seeded into 75-cm^2^ flasks at a density of approximately 1 × 10^6^ cells per flask and maintained at 37 °C in a humidified atmosphere of 5 % CO_2_ in air in EGM™ endothelial growth medium supplemented with EGM™-2 BulletKit™. All tissue culture reagents were supplied by Lonza. The medium was replaced twice weekly. Experiments were carried out at passage 5–6 in EGM™ endothelial growth medium supplemented with 2 % (*v*/*v*) fetal calf serum (Sigma) and 60 μg/ml gentamycin (Sigma).

### Overexpression and knockdown of SIRT1

Overexpression of SIRT1 in both Caco-2 cells and HuVECs was achieved by transient transfection with the plasmid pCMV6-ENTRY-SIRT1 (Origene), and knockdown of SIRT1 was achieved using two different siRNAs and compared with a control siRNA, as described previously for Caco-2 cells [16]. Efficacy of overexpression and knockdown was confirmed by RT-qPCR and Western blotting for both cell lines as described previously for Caco-2 cells [16].

### Preparation of DNA and enrichment for the methylated fraction

DNA was extracted from HuVECs and Caco-2 cells using the QIAamp® DNA Mini Kit (Qiagen). Enrichment of HuVEC DNA for the methylated fraction was carried out by NXT-DX, The Netherlands. DNA was fragmented to an average length of 200 bp by sonication and analysed using the Agilent 2100 Bioanalyser (Agilent Technologies). Methylated DNA was captured using the MethylCap kit (Diagenode), based on binding to methylated DNA of a recombinant H6-GST-MBD protein then captured on magnetic beads coated with GSH. Caco-2 DNA was fragmented by sonication and purified from agarose gels to produce DNA fragments ranging from 200–1000 bp in length. Purified fragmented DNA was then spiked with unmethylated and methylated internal controls generated from Lambda phage genomic DNA. To generate these samples, two different fragments of ~500 bp in length were amplified by PCR from Lambda phage dam^−^ dcm^−^ genomic DNA (Fermentas) using high specificity HotStar Taq DNA polymerase (Qiagen) and primer pairs AGCAACCAACAAGAAAACACT plus TCATCCTCGGCAAACTCTTT and GTGAGGTGAATGTGGTGAAGT plus TCGCAGAGATAAAACACGCT. An aliquot of each PCR product was then methylated in vitro using *SssI* DNA methylase (New England Biolabs). The methylation status of the unmethylated and methylated controls was confirmed by digestion with the methylation-sensitive restriction enzyme *AciI* (Fermentas). Spiked DNA samples were denatured at 95 °C then incubated for 4 h at 4 °C with anti-5-methylcytidine (5mC) antibody (Eurogentec) (12 μl per 4.5 μg DNA) in 10 mM Na-Phosphate pH 7.0, 140 mM NaCl, 0.05 % Triton X-100. DNA was then captured using sheep anti-mouse IgG Dynabeads (Invitrogen), following the manufacturer’s instructions. Dynabeads were then incubated with Proteinase K (Roche) at 50 °C for 2 h with shaking, and the beads were then removed using a magnetic rack. DNA was then purified using the DNA Clean and Concentrator 5 kit (Zymo Research). Whole genome amplification of input or immunoprecipitated DNA was then performed using the GenomePlex® Complete Whole Genome Amplification kit (Sigma), and retention of fragment size was confirmed by agarose gel electrophoresis.

### Analysis of differences in the DNA content of input versus enriched pools

For each condition (SIRT1 overexpressed by transfection with pCMV6-ENTRY-SIRT1, corresponding vector control, each of the siRNAs that target SIRT1 and control siRNA), six biological replicates prepared from HuVECs were pooled for analysis. Sequence reads were mapped to hg19 using Bowtie2 [42] then filtered first to remove un-mapped reads using Picard (http://picard.sourceforge.net/). The GENCODE gene set [43] was then used to identify genes, defined as within 2 kb of a TSS or within genes (between the TSS and stop codon). The package MEDIPS (Bioconductor) was used to identify differentially methylated genes, which were then classified as hypomethylated or hypermethylated when SIRT1 expression was increased or reduced.

The methylation profile of DNA from Caco-2 cells was measured using the DNA Methylation Service provided by NimbleGen. Input and methylation-enriched samples were labelled with Cy3 and Cy5, respectively, using the NimbleGen Dual-Colour DNA Labelling Kit then co-hybridised to the Human 3x720K CpG Island Plus RefSeq Promoter Array using the NimbleGen Hybridisation System. The hybridised arrays were then washed with the NimbleGen Wash Buffer Kit and dried with the NimbleGen Microarray Drier, and signal peak intensities for each probe were measured with the NimbleGen MS 200 Microarray Scanner. Data were provided as signal intensity and *P* value for each probe. These data were used to detect peaks through manual inspection in Excel (Microsoft), accepting positive enrichment being where at least two of three biological replicates of each sample analysed (SIRT1 overexpressed by transfection with pCMV6-ENTRY-SIRT1, corresponding vector control, each of the siRNAs that target SIRT1 and control siRNA) met a >2 *P* value minimum cutoff.

### RNA measurement by RT-qPCR

RNA was prepared using the PureLink RNA MiniKit (Life Technologies) then reverse transcribed using SuperScript III Reverse Transcriptase (Life Technoloiges), following the manufacturer’s instructions. Quantitative real-time PCR was performed in a Roche LightCycler 480 with 20 μl reactions set up in a 96-well format containing LightCycler SYBR Green I Master (Roche), 0.5 μM of each primer (see Additional file 2) and 1 μl of cDNA (reverse transcription reaction diluted 1:4). After denaturing for 5 min at 95 °C, 50 cycles were carried out using the following parameters: 95 °C, 10 s; 55 °C, 10 s; and 72 °C, 15 s. Levels of specific RNAs relative to control, and corrected according to levels of reference gene RNAs, were calculated using the ΔΔC_t_ method. PCR products were sequenced (Eurofins Genomics) to confirm identity.

### Immunoprecipitation

SIRT1 or EZH2 and proteins bound to them were co-immunoprecipitated using the Classic IP kit (Pierce). Cells were rinsed with 1× PBS and lysed with lysis/wash buffer in volumes according to cell growth area, as recommended by the manufacturer. Lysate samples (1000 μg each) were pre-cleared with control agarose resin, followed by overnight incubation at 4 °C with either 10 μg SIRT1 antibody (Abcam, ab7343) or 10 μg EZH2 antibody (R&D Systems, AF4767). Negative controls were prepared in an identical manner but omitting antibody. Immune complexes were immobilised on protein A/G Sepharose columns, which were washed three times with lysis/wash buffer then once with conditioning buffer. Protein was then eluted in elution buffer and concentration was determined against BSA standards using Bradford reagent (Bio-Rad Laboratories).

### Western blotting

Immunoprecipitated proteins or total cell protein extract prepared as described previously [16] (10 μg) were resolved by SDS-PAGE using 7 % gels then transferred by semi-dry blotting onto PVDF membrane (Amersham Hybond-P, GE Healthcare). Membranes were incubated at room temperature for 1 h with constant shaking first in Odyssey blocking buffer (LI-COR) then with primary antibody (SIRT1 (Abcam, ab7343) or EZH2 (R&D Systems, AF4767 )) diluted 1:250 in Odyssey blocking buffer supplemented with 0.1 % (*v*/*v*) Tween-20. Membranes were washed five times for 15 min in 1× PBS plus 0.1 % (*v*/*v*) Tween-20 then incubated for 1 h at room temperature with secondary antibody (Odyssey LI-COR, IRDye® 680RD donkey anti-rabbit IgG to detect anti-SIRT1 and IRDye® 800CW donkey anti-goat IgG to detect EZH2) diluted 1:2000 in Odyssey blocking buffer supplemented with 0.1 % (*v*/*v*) Tween-20. The washing procedure was then repeated before scanning membranes on the Odyssey LI-COR infrared imaging system.

### Statistical analysis

Intersections between gene lists were tested for statistical significance by chi-square analysis applying Yates’ correction. Data derived by RT-qPCR were analysed by one-way ANOVA then Dunnett’s post hoc test.

### Availability of supporting data

The data sets supporting the results of this article are available in the Gene Expression Omnibus database (http://www.ncbi.nlm.nih.gov/geo/query/acc.cgi?acc=GSE54072 and http://www.ncbi.nlm.nih.gov/geo/query/acc.cgi?acc=GSE53569).

## Additional files

Additional file 1:
**Enrichment ratios of methylated DNA after MeDIP compared to input DNA for Caco-2 cells.**
Additional file 2:
**Primers used for qPCR.**

## Abbreviations

MeDIP: methylated DNA immunoprecipitation
DNMT: DNA methyltransferase
PCGT: polycomb group protein target gene
PRC: polycomb repressive complex
RF: representation factor
SIRT1: sirtuin 1
TSS: transcription start site
