# Supplementary material for: SIRT1 affects DNA methylation of polycomb group protein target genes, a hotspot of the epigenetic shift observed in ageing
Source: Hum Genomics. 2015 Jun 24;9(1):14. doi: 10.1186/s40246-015-0036-0 (PMC4480908; doi:10.1186/s40246-015-0036-0)
Supplement: Additional file 1: — Enrichment ratios of methylated DNA after MeDIP compared to input DNA for Caco-2 cells. [file 40246_2015_36_MOESM1_ESM.docx]

Enrichment ratios of methylated DNA after MeDIP compared to input DNA for Caco-2 cells. Enrichment was calculated for each sample using C_t_ values for negative controls subtracted from values for samples. C_t_ values for immunoprecipitated samples were subtracted from input values (X) and enrichment expressed as 2X. Data are for two biological replicates prepared and analysed for each experimental manipulation.

|  | Vector control | | pCMV6-ENTRY-SIRT1 | | Control siRNA | | SIRT1 siRNA1 | | SIRT1 siRNA2 | |
| --- | --- | --- | --- | --- | --- | --- | --- | --- | --- | --- |
|  | 1 | 2 | 1 | 2 | 1 | 2 | 1 | 2 | 1 | 2 |
| Lambda phage spike | 2200 | 9100 | 12000 | 9000 | 1000 | 2000 | 12000 | 7000 | 4200 | 3700 |
| H19/UBE2B | 110 | 270 | 70 | 260 | 140 | 120 | 60 | 70 | 170 | 110 |
| L1.2/UBE2B | 120 | 250 | 40 | 300 | 100 | 70 | 100 | 40 | 160 | 160 |
