# Supplementary material for: SIRT1 affects DNA methylation of polycomb group protein target genes, a hotspot of the epigenetic shift observed in ageing
Source: Hum Genomics. 2015 Jun 24;9(1):14. doi: 10.1186/s40246-015-0036-0 (PMC4480908; doi:10.1186/s40246-015-0036-0)
Supplement: Additional file 2: — Primers used for qPCR. [file 40246_2015_36_MOESM2_ESM.docx]

Primers used for qPCR. All sequences are in the 5’-3’ direction.

| **Amplicon** | **Primers (5’-3’)** |
| --- | --- |
| SIRT1 | GGCAAAGGAGCAGATTAGTAGG  CATCAGGCTCATCCTTCTAAGCC |
| GAPDH | TGAAGGTCGGAGTCAACGGATTTG  CATGTAAACCATGTAGTTGAGGTC |
| TOP1 | GCGAACTTAGGCTGT  AATCGTTGTGGAGGTGG |
| RbAp48 | CAGTGGAAGAACGAGTGATC  GCATTCA TCGACTTGTCC |
| EED | GGAATATCCAGACGGACACTC  GAGAATGATCCATACCACAGG |
| SUZ12 | GTGATACCTGCTTACCTCTCC  CATGACATGGAGATTCCAG |
| EZH2 | GTCCTCATTGGCACTTACTATG  CTTGGAGGAGTATCCACATC |
| BMI1 | GCAGCTCGCTTCAAGATG  GAGGGTACTTCATTGATGCC |
| PHC1 | CAGTGCTACCACCTTGAC  AGGAGACTGAGCAGATGG |
| RNF2 | GATGACAGTGCACAGACGAG  GTATACTGCTTCTCACTGGCTG |
| KDM2B | GGACATCACAGATGCCTC  CTTATTGCAGTCAGACAGGTTG |
| DNMT3b | GAAGACTCGATCCTCGTC  GTGTCCAGTCTGCTAAGCTAC |
| DNMT1 | GAGCTGAACCTTCACCTAGC  GGATGAGTCCATCAAGGAAG |
